# Circulating immune complexes in dogs with osteosarcoma.

**DOI:** 10.1038/bjc.1982.223

**Published:** 1982-09

**Authors:** A. Segal-Eiras, R. A. Robins, D. Hannant, L. N. Owen, R. W. Baldwin


*Br. J. Cancer* (1982) **46**, 444

***Short Communication***

### CIRCULATING IMMUNE COMPLEXES IN DOGS WITH OSTEOSARCOMA

A. SEGAL-EIRAS*, R. A. ROBINS*, D. HANNANT*†, L. N. OWEN† AND R. W. BALDWIN*

*From the *Cancer Research Campaign Laboratories, University of Nottingham, and †Department of Clinical Veterinary Medicine, University of Cambridge, Cambridge*

Received 4 January 1982 Accepted 30 April 1982

CANCER HAS become an increasingly important disease in canine populations, partly as a result of advances in veterinary medicine, where elimination of many infectious diseases by vaccination has considerably extended the average life-span of dogs. Deaths are now more frequently attributed to diseases of old age, of which cancer is an imporant example (Hannant *et al.*, 1978).

Many diseases of dogs present a similar clinico-pathological picture to their counterparts in humans, and there are several comparative studies relating clinical and morphological aspects of canine tumours. There are, however, few reports existing on the adaptability of classic markers of disease. A study of the comparative pathology between human and canine osteosarcoma (Owen, 1969) showed that the biological behaviours of this tumour is similar in the two species. Other neoplasms of comparative medical and veterinary interest include spontaneous mammary carcinoma (Owen, 1979) where circulating immune complexes have been demonstrated.

Immune complexes have been studied in relation to the pathogenesis of human and animal disorders including malignant diseases (Höffken *et al.*, 1978*b*; Baldwin *et al.*, 1979; Terman *et al.*, 1980). In recent years sensitive techniques have been applied to detect immune complexes in sera from patients with many types of tumour (reviewed by Baldwin & Robins, 1980). Circulating immune complexes in human osteosarcoma have been studied using the Clq binding test by Tsang *et al.* (1979) and in this laboratory by Segal-Eiras *et al.* (1980).

The possibility of investigating a large population of dogs with o.s. allowed the development of the present study, in which the incidence of sera with raised Clq binding could be determined, and a preliminary investigation of changes in Clq binding with disease progress could be made.

These investigations were performed in parallel with human bone-tumour immune-complex studies, and used human Clq, which has previously been shown to bind rat (Hoffken *et al.*, 1978*a*) and dog (Terman *et al.*, 1979) immune complexes. Following the method of Yonemasu & Stroud (1971) Clq was prepared and purified from pooled normal human sera and its purity was checked by immunoelectrophoretic analysis. The Clq preparation was radioiodinated with lactoperoxidase (Heusser *et al.*, 1973) to give a sp. act. of $\sim 1 \mu Ci/\mu g$. All studies were performed with $^{125}I$ labelled Clq stored no longer than 15 days at $-70°C$. Immune complexes were measured by $^{125}I$-Clq binding test, following the method of Zubler *et al.* (1976) essentially as previously described (Segal-Eiras *et al.*, 1980).

The baseline serum Clq-binding levels

Present address: Institute of Occupational Medicine, City Hospital, Greenbank Drive, Edinburgh.

TABLE I.—*Serum $^{125}$I-Clg binding levels in dogs with osteosarcoma, other tumours and healthy controls*

| Serum samples | Clg binding* (mean ± s.d.) | Range | Number +ve† | % |
|---|---|---|---|---|
| Dog osteosarcoma | 39·9 ± 14·7 | 13·1–64·4 | 46/56 | 82·1 |
| Other tumours | 34·4 ± 14·2 | 21·9–64·3 | 10/10 | 100 |
| Non-malignant diseases | 11·2 ± 3·0 | 7·5–14·8 | 1/8 | 13·0 |
| Healthy control | 8·5 ± 2·7 | 5·5–12·3 | 0/34 | 0 |

* 4–5 tests for each serum sample.
† > Mean + 2 s.d. of healthy dogs group (14% Clg binding).

TABLE II.—*Protein A affinity of Clq binding in serum of dogs with osteosarcoma*

| | | % Clq binding (mean ± s.e.) | | |
|---|---|---|---|---|
| Sera | Test | Before separation | Protein A unbound | Bound and eluted |
| Osteosarcoma 1 | 1 | 19·9 ± 0·2 | 5·2 ± 0·1 | 26·5 ± 1·5 |
| | 2 | 28·8 ± 0·5 | 4·5 ± 0·2 | 32·2 ± 2·9 |
| Osteosarcoma 2 | 1 | 18·3 ± 1·1 | 5·6 ± 0·1 | 19·7 ± 0·5 |
| Osteosarcoma (pool) | 1 | 26·1 ± 1·9 | 3·0 ± 0·5 | 25·8 ± 0·2 |
| | 2 | 31·5 ± 2·0 | 2·5 ± 0·4 | 29·2 ± 0·4 |
| Normal dog (pool 1) | 1 | 6·2 ± 0·5 | 24·9 ± 0·7 | 5·0 ± 0·1 |
| | 2 | 4·7 ± 0·6 | 18·8 ± 1·5 | 2·6 ± 0·1 |
| Normal dog (pool 2) | 1 | 9·1 ± 0·3 | 23·5 ± 0·3 | 5·3 ± 0·3 |

Serum samples were applied to Sepharose 4B-CL Protein A columns. Unbound protein eluted in PBS and bound fractions eluted with pH 2·8 buffer were neutralized, concentrated to the volume of the original serum sample, dialysed against PBS and tested in the $^{125}$I-Clq binding assay.

for normal dogs was established in tests of samples from 22 clinically healthy female beagles aged 2–7 years, and 12 healthy wolfhounds of both sexes with an age range of 5–7 years. Clq-binding values shown in Table I ranged from 5·5 to 12·3% (mean 8·5 ± 2·7%). For comparative purposes, a high value was taken to be > 14·0% or mean + 2 s.d. of the control serum samples. Fifty-six dogs with spontaneous osteosarcoma confirmed at biopsy or post mortem examination were studied; most were of large breed (Owen, 1969). Forty-six out of 56 serum samples from dogs with osteosarcoma had high Clq-binding levels ranging from 14·2 to 54·4 (mean 39·9 ± 14·7%).

Sera from dogs with either lymphosarcoma, fibrosarcoma or mammary carcinoma also had high levels in 10/10 cases; range 21·9–64·3 (mean 34·4 ± 13·4). In contrast, sera from a small group of dogs with non-malignant diseases, including healing fractures, osteoarthritis, diabetes melitus, otitis externa and skin diseases, had low levels of Clq binding (1/8 positive, range 7·5–14·8, mean 11·2 ± 3·0).

Experiments were performed to investigate the nature of the serum Clq binding material by determining its affinity for Protein A. Serum samples were dialysed against buffered saline (PBS pH 7·2) and applied to a 2ml column of Protein A-Sepharose CL4B (Pharmacia) equilibrated with the same buffer. Unbound material was eluted with PBS, and bound fractions eluted with 0·1M glycine HCl buffer (pH 2·8). After neutralization, serum fractions were concentrated to the original volume, dialysed against PBS and centrifuged (800 *g*) for 30 min before testing in the Clq-binding assay. Because of the limited quantities of sera available, these studies were performed using not only individual samples but also from a pool of sera from 3 dogs with o.s. (Table II). With o.s. samples, the fractions which bound to and eluted from Protein A immobilized on Sepharose CL-4B gave Clq binding, comparable with that in the unfractionated

FIG. 1.—Sequential study of serum Clq binding levels in 4 dogs with osteosarcoma without recurrence after one year. Dog C was amputated and treated with BCG. Dogs F, H and K received radiotherapy (10 Gy from a linear accelerator to the affected bone at weekly intervals, to a total dose of 40 Gy).

FIG. 2.—Sequential study of serum Clq binding levels in 8 dogs with osteosarcoma with local recurrence and/or metastasis. Dogs A and B were amputated and irradiated. Dogs D, E, G, I and L received radiotherapy. Dog J was amputated at diagnosis followed by BCG and chemotherapy.

sample. Clq binding of the Protein A-unbound material was normal, both in the individual dogs with o.s. and the pooled o.s. serum samples. The Protein A bound and eluted fractions from two pools of normal dog sera showed low Clq binding, though the Protein A-unbound fractions of these sera showed anomalous high Clq binding. The basis of Clq binding from these normal sera during fractionation is not known.

Sequential serum samples were available from 12 dogs in this study. The results of Clq-binding assays on sera from 4 dogs surviving up to 1 year without recurrences are shown in Fig. 1. This group of animals had high Clq-binding levels before treatment, which returned to the normal range by 1 year. A further 8 dogs were studied (Fig. 2) in which treatment was unsuccessful. In these cases, Clq binding was high at diagnosis, and remained high throughout the disease course, or fell only transiently. The demonstration of high Clq binding in the serum of dogs with osteosarcoma, and its relationship to the course of disease, show similarities to earlier studies in human osteosarcoma (Segal-Eiras *et al.*, 1980). One value of these sequential studies lies in the fact that the canine osteosarcoma has a more rapid course than in man, and results on the effect of therapy and monitoring of the disease can be obtained sooner.

Studies on the Protein A-Sepharose affinity of osteosarcoma and normal dog sera showed that in osteosarcoma sera, the Clq binding appeared in the bound and low pH-eluted fraction. On the contrary, the values of Clq binding of the same fraction from normal dogs were low. These results suggest that the immune complexes detected with this test have components with IgG characteristics, though the anomalous Clq binding in the unbound fraction of normal dog sera means that these results must be regarded with caution.

The investigations show the adaptability of a human clinical method to a canine system and further studies will be necessary to resolve the nature of these immune complexes in canine osteosarcoma.

This work was supported by the Cancer Research Campaign. A. Segal-Eiras received additional support from CONICET (Consejo Nacional de Investigaciones Cientificas y Tecnicas) Buenos Aires, Argentina.

### REFERENCES

BALDWIN, R. W., BYERS, V. S. & ROBINS, R. A. (1979) Circulating immune complexes in cancer: Characterization and potential as tumour markers. *Behring Inst. Mitt.*, **64**, 63.

BALDWIN, R. W. & ROBINS, R. A. (1980) Circulating immune complexes in cancer. In *Cancer Markers: Developmental and Diagnostic Significance* (Ed. Sell) Clifton N.J.: Humana Press. p. 507.

HANNANT, D., ELSE, R. W. & CRIGHTON, G. W. (1978) Antigens associated with canine spontaneous mammary carcinoma. *Vet. Rec.*, **103**, 441.

HEUSSER, C., BOESMAN, M., NORDIN, J. H. & ISLIKER, H. (1973) Effect of chemical and enzymatic radioiodination on *in vitro* human Clq activities. *J. Immunol.*, **110**, 820.

HÖFFKEN, K., MCLAUGHLIN, P. J., PRICE, M. R., PRESTON, V. E. & BALDWIN, R. W. (1978*a*) Rat Clq: Similarity to human Clq in functional and composition properties. *Immunochemistry*, **15**, 409.

HÖFFKEN, K., PRICE, M. R., MOORE, V. E. & BALDWIN, R. W. (1978*b*) Circulating immune complexes in rats bearing chemically induced tumours. II. Characterization of sera from different stages of tumour growth. *Int. J. Cancer*, **22**, 576.

OWEN, L. N. (1969) *Bone Tumours in Man and Animals*. London: Butterworth & Co.

OWEN, L. N. (1979). A comparative study of canine and human breast cancer. *Invest. Cell. Pathol.*, **3**, 257.

SEGAL-EIRAS, A., ROBINS, R. A., BALDWIN, R. W. & BYERS, V. S. (1980) Circulating immune complexes in patients with bone tumours. *Int. J. Cancer*, **25**, 735.

TERMAN, D. S., MOORE, D., COLLINS, J. & 5 others (1979) Detection of immune complexes in the sera of dogs with rheumatic and neoplastic diseases by $^{125}$I-Clq binding test. *J. Comp. Pathol.*, **89**, 221.

TERMAN, D. S., YAMAMOTO, T., MATTIOLI, M. & 5 others (1980) Extensive necrosis of spontaneous canine mammary adenocarcinoma after extracorporeal perfusion over *Staphylococcus aureus* Cowan I. *J. Immunol.*, **124**, 795.

TSANG, K. T., SINGH, I. & BLADEMORE, W. S. (1979) Circulating immune complexes in human osteosarcoma. *J. Natl Cancer Inst.*, **62**, 743.

YONEMASU, K. & STROUD, R. N. (1971) Clq: Rapid purification method for preparation of monospecific antisera and biochemical studies. *J. Immunol.*, **106**, 304.

ZUBLER, R. H., LANGE, G., LAMBERT, P. J. & MIESCHER, P. A. (1976) Detection of immune complexes in unheated sera by a modified $^{125}$I-Clq binding test. *J. Immunol.*, **116**, 232.


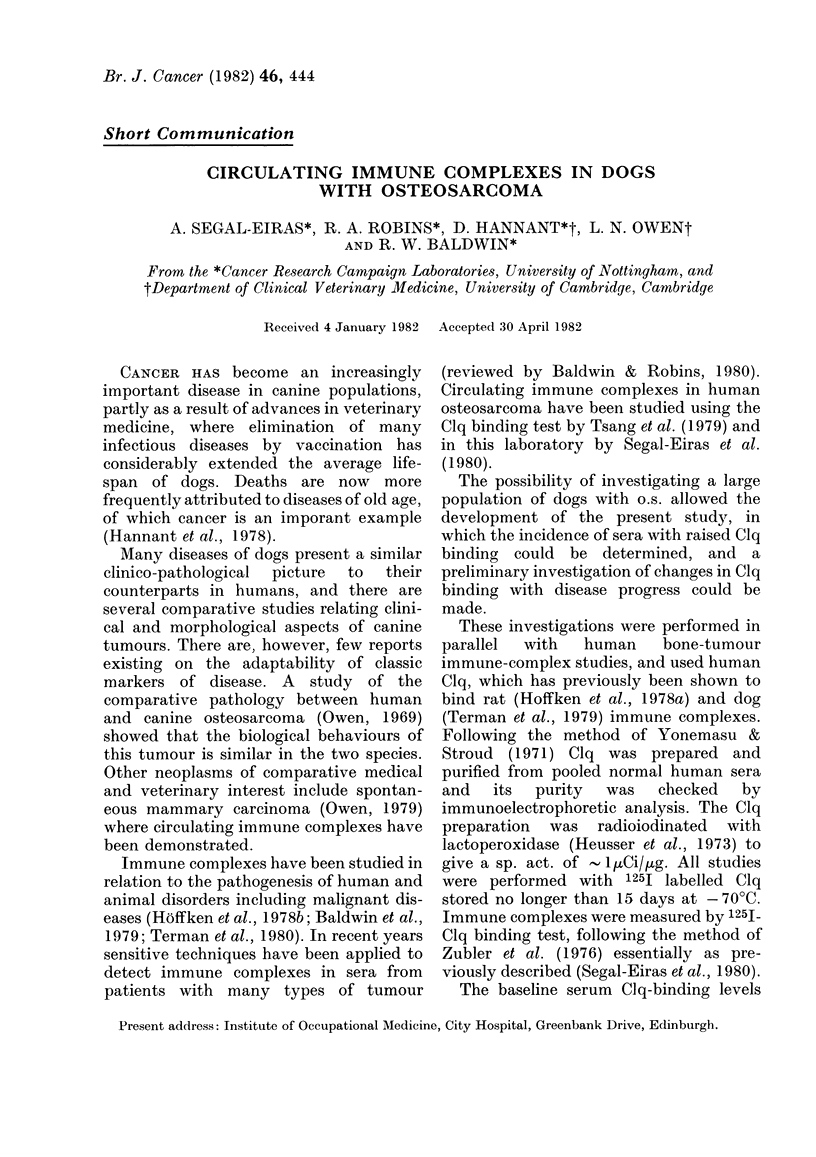

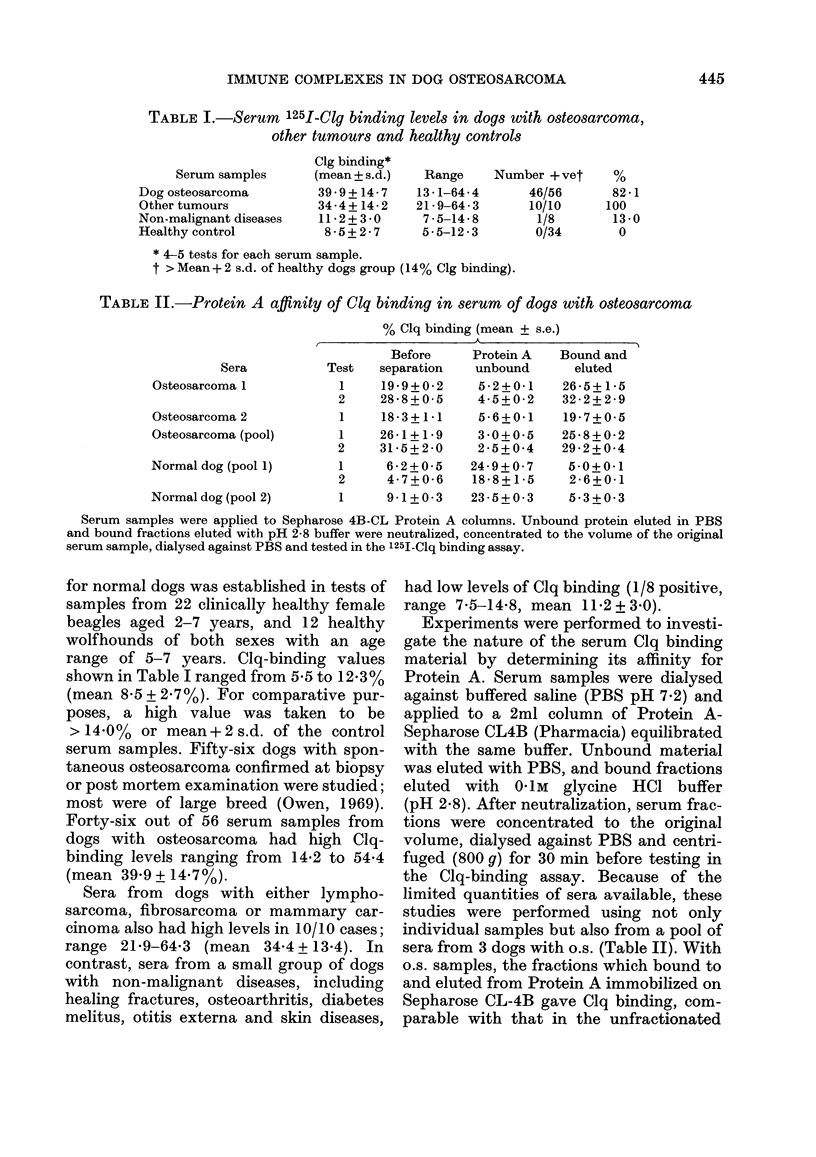

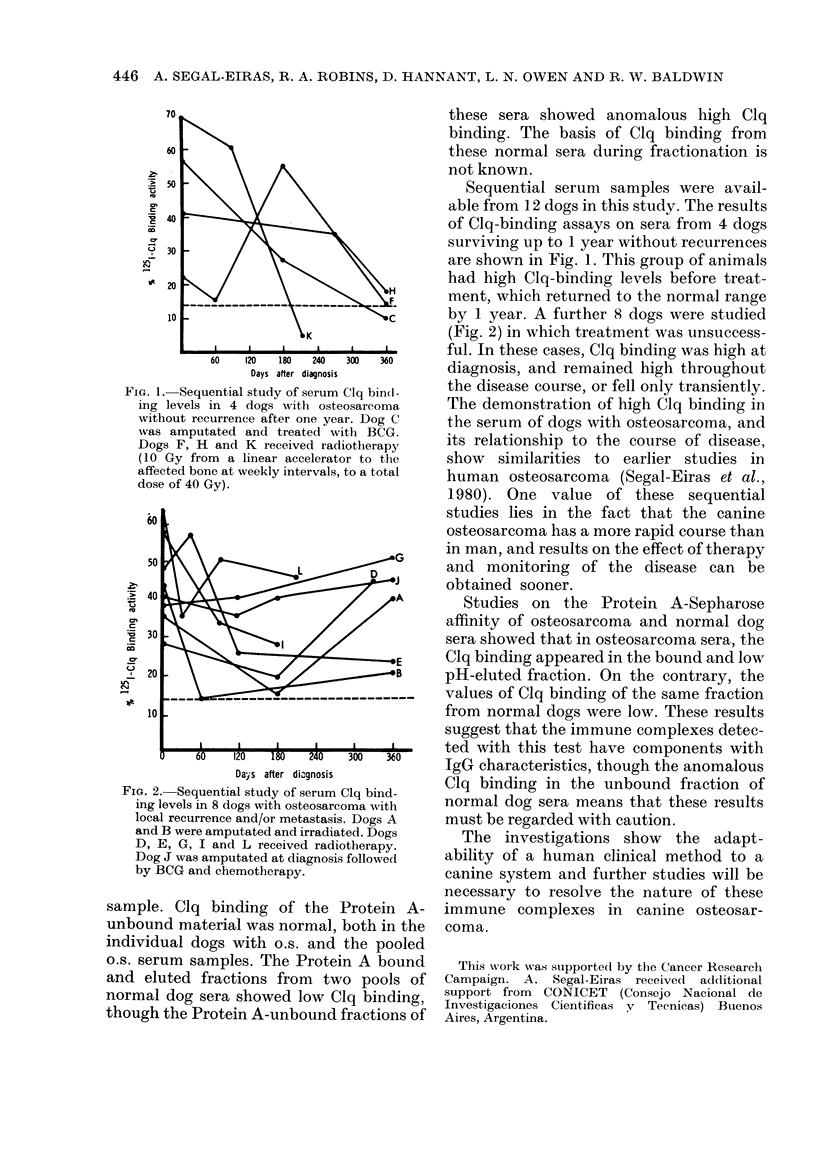

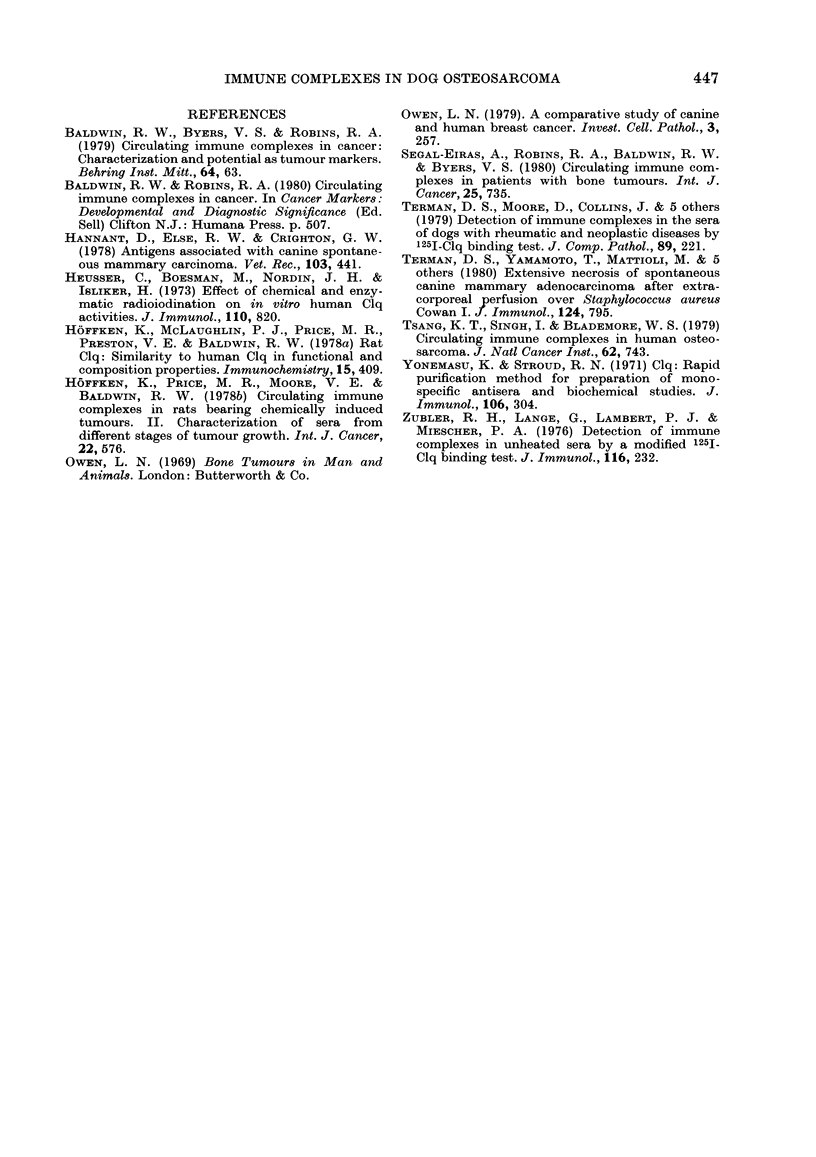


## References

[OCR_00414] Eiras A. S., Robins R. A., Baldwin R. W., Byers V. S. (1980). Circulating immune complexes in patients with bone tumours.. Int J Cancer.

[OCR_00378] Hannant D., Else R. W., Crighton G. W. (1978). Antigens associated with canine spontaneous mammary carcinoma.. Vet Rec.

[OCR_00383] Heusser C., Boesman M., Nordin J. H., Isliker H. (1973). Effect of chemical and enzymatic radioiodination on in vitro human Clq activities.. J Immunol.

[OCR_00391] Höffken K., McLaughlin P. J., Price M. R., Preston V. E., Baldwin R. W. (1978). Rat Clq: similarity to human Clq in functional and compositional properties.. Immunochemistry.

[OCR_00396] Höffken K., Price M. R., Moore V. E., Baldwin R. W. (1978). Circulating immune complexes in rats bearing chemically induced tumours. II. Characterization of sera from different stages of tumour growth.. Int J Cancer.

[OCR_00408] Owen L. N. (1979). A comparative study of canine and human breast cancer.. Invest Cell Pathol.

[OCR_00417] Terman D. S., Moore D., Collins J., Johnston B., Person D., Templeton J., Poser R., Quinby F. (1979). Detection of immune complexes in sera of dogs with rheumatic and neoplastic diseases by 125I-Clq binding test.. J Comp Pathol.

[OCR_00423] Terman D. S., Yamamoto T., Mattioli M., Cook G., Tillquist R., Henry J., Poser R., Daskal Y. (1980). Extensive necrosis of spontaneous canine mammary adenocarcinoma after extracorporeal perfusion over Staphylococcus aureus Cowans I. I. Description of acute tumoricidal response: morphologic, histologic, immunohistochemical, immunologic, and serologic findings.. J Immunol.

[OCR_00430] Tsang K. Y., Singh I., Blakemore W. S. (1979). Circulating immune complexes in human osteosarcoma.. J Natl Cancer Inst.

[OCR_00435] Yonemasu K., Stroud R. M. (1971). Clq: rapid purification method for preparation of monospecific antisera and for biochemical studies.. J Immunol.

[OCR_00441] Zubler R. H., Lange G., Lambert P. H., Miescher P. A. (1976). Detection of immune complexes in unheated sera by modified 125I-Clq binding test. Effect of heating on the binding of Clq by immune complexes and application of the test to systemic lupus erythematosus.. J Immunol.

